# Sevoflurane Preconditioning Reduces Intestinal Ischemia-Reperfusion Injury: Role of Protein Kinase C and Mitochondrial ATP-Sensitive Potassium Channel

**DOI:** 10.1371/journal.pone.0141426

**Published:** 2015-10-27

**Authors:** Chuiliang Liu, Yanhui Liu, Zhiwen Shen, Liping Miao, Kun Zhang, Fei Wang, Yujuan Li

**Affiliations:** 1 Department of Anesthesiology, ChanCheng Center Hospital, Foshan, Guangdong, China; 2 Department of Anesthesiology, Sun Yat-sen Memorial Hospital, Sun Yat-sen University, Guangzhou, Guangdong, China; University of Colorado Denver, UNITED STATES

## Abstract

Ischemic preconditioning (IPC) has been considered to be a potential therapy to reduce ischemia-reperfusion injury (IRI) since the 1980s. Our previous study indicated that sevoflurane preconditioning (SPC) also reduced intestinal IRI in rats. However, whether the protective effect of SPC is similar to IPC and the mechanisms of SPC are unclear. Thus, we compared the efficacy of SPC and IPC against intestinal IRI and the role of protein kinase C (PKC) and mitochondrial ATP-sensitive potassium channel (mK_ATP_) in SPC. A rat model of intestinal IRI was used in this study. The superior mesenteric artery (SMA) was clamped for 60 min followed by 120 min of reperfusion. Rats with IPC underwent three cycles of SMA occlusion for 5 min and reperfusion for 5 min before intestinal ischemia. Rats with SPC inhaled sevoflurane at 0.5 minimum alveolar concentration (MAC) for 30 min before the intestinal ischemic insult. Additionally, the PKC inhibitor Chelerythrine (CHE) or mK_ATP_ inhibitor 5-Hydroxydecanoic (5-HD) was injected intraperitoneally before sevoflurane inhalation. Both SPC and IPC ameliorated intestinal IRI-induced histopathological changes, decreased Chiu’s scores, reduced terminal deoxyribonucleotide transferase-mediated dUTP nick end labeling (TUNEL) positive cells in the epithelium, and inhibited the expression of malondialdehyde (MDA) and tumor necrosis factor-α (TNF-α). These protective effects of SPC were similar to those of IPC. Pretreatment with PKC or mK_ATP_ inhibitor abolished SPC—induced protective effects by increasing Chiu’s scores, down-regulated the expression of Bcl-2 and activated caspase-3. Our results suggest that pretreatment with 0.5 MAC sevoflurane is as effective as IPC against intestinal IRI. The activation of PKC and mK_ATP_ may be involved in the protective mechanisms of SPC.

## Introduction

Intestinal ischemia-reperfusion injury (IRI) is a potentially severe consequence of several surgical procedures, including abdominal aortic aneurysm surgery, cardiopulmonary bypass, intestine transplantation and strangulated hernias [1, 2]. Mucosal barrier failure, bacteria translocation and systemic inflammation play critical roles in the pathophysiology of intestinal IRI, which may even result in multi-organ dysfunction syndrome (MODS) [1, 2]. Considering the disastrous outcomes of intestinal IRI, more efforts are needed to develop effective and safe therapeutic methods.

A brief episode of artificial ischemia prior to subsequent ischemic insults is known as ischemic preconditioning (IPC). Intestinal IPC has been identified as an effective measure to reduce intestinal IRI [3–8]. However, IPC has limited application in the clinic due to its invasive nature and the unpredictability of when intestinal ischemia will occur. Our recent finding suggested that sevoflurane, a widely used volatile anesthetic, reduces IR—induced intestinal injury at clinical related concentrations when given before, during or after ischemia. Moreover, sevoflurane preconditioning (SPC) at 0.5 minimum alveolar concentration (MAC) is the most effective method among all the strategies [9]. However, whether the protective effect of SPC is similar to IPC is still unclear. A comparison between these two interventions will help to choose a more appropriate therapeutic method if intestinal IRI is inevitable.

An increasing amount of studies have reported that sevoflurane protects against ischemia-reperfusion injury of multiple organs [10–14]. The protective mechanisms include amelioration of apoptosis [13] and reduction of oxidative stress and inflammation response [12, 14, 15]. Our recent study also demonstrated that pretreatment with sevoflurane reduces intestinal IRI and apoptosis via activation of the phosphatidylinositol 3 kinases (PI3K) / Akt pathway. However, the PI3K inhibitor only partly reverses the protection of sevoflurane, suggesting that other mechanisms may be involved. Protein kinase C (PKC) and mitochondrial ATP-sensitive potassium channel (mK_ATP_) are important components of the intracellular signaling pathway [16, 17]. It has been demonstrated that activation of PKC epsilon (PKCε) and mK_ATP_ play important roles in the cardioprotection of isoflurane preconditioning and arginase inhibition in myocardium IRI [18, 19]. PKC and mK_ATP_ pathways are also involved in the delayed neuroprotection of SPC [20]. Moreover, activation of nPKC isoform(s), especially PKCε and KATP channels may have an important role in the protective effects of IPC against intestinal IRI [21, 22]. However, whether SPC-induced reduction of intestinal IRI is directly associated with activation of PKC and mK_ATP_ pathways remains to be clarified.

Therefore, the aim of the present study is to compare the protection of IPC and SPC in rats with intestinal IR. Furthermore, we tested the hypothesis that SPC provides protection against intestinal IRI by activating PKC and opening the mK_ATP_ channels.

## Materials and Methods

All experimental procedures and protocols in this study were approved by the animal care committee at Sun Yat-sen University, Guangzhou, China and were performed in strict accordance with the National Institutes of Health Guidelines for the use of experimental animals. All efforts were made to minimize suffering of the animals.

Adult male Sprague-Dawley rats weighing 200–220 g were obtained from Guangdong Medical Laboratory Animal Co, China (Permission number: SCXK 2008–0002). They were housed under standardized conditions of temperature (22°C- 25°C), humidity (55%–58%), and 12-h dark-light cycle with free access to food and water. Animals were starved for 12 h prior to the experimentation, but allowed free access to water. Seventy-two rats in total were enrolled in this study.

### Model of intestinal IRI

The rat model of intestinal IRI was established as we previously described [9]. Briefly, the rats were anesthetized by intraperitoneal injection of 20% urethane (0.6 ml/100g) and allowed to breath spontaneously during the surgery. The superior mesenteric artery (SMA) was isolated and occluded with a non-invasive artery clamp. Intestinal ischemia was identified by the loss of mesenteric pulsation and paleness of the intestine. The intestine was put back into the abdominal cavity in situ. After 60 min of intestinal ischemia, the clamp was removed from the SMA, and reperfusion lasted for 120 min. The reperfusion was verified by the recovery of mesenteric pulsation and a flush of the intestine. The sham group rats underwent laparotomy without SMA occlusion. For intestinal IPC treatment, rats underwent SMA occlusion for 5 min and then reperfusion for 5 min for three cycles prior to the subsequent treatment.

### Experimental protocol

**Fig 1:**
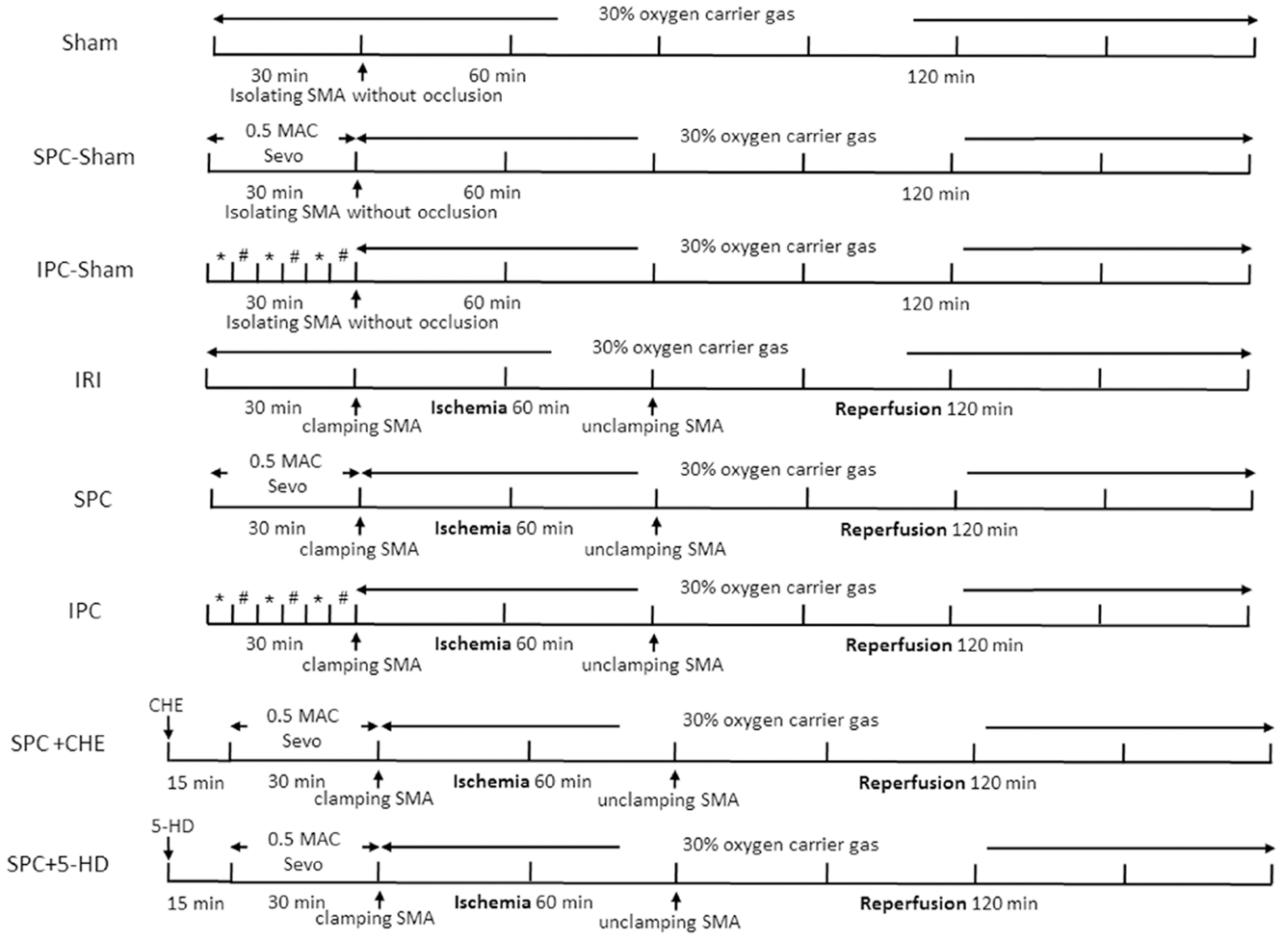
The protocol of the experiment one and two. Sham: involving isolation of SMA without occlusion; IRI: performed SMA occlusion for 60 min followed by reperfusion for 120 min without any interventions; SPC-sham: Rats were pretreated with 0.5 MAC sevoflurane for 30 min before sham operation; IPC-sham: Rats underwent intestinal IPC without 60 min ischemia; SPC: Rats were pretreated with 0.5 MAC sevoflurane for 30 min before 60 min ischemia; IPC: Rats underwent intestinal IPC prior to 60 min ischemia; SPC + CHE: Rats received intraperitoneal injection of the PKC inhibitor Chelerythrine (5 mg/kg) 15 min before SPC; SPC + 5-HD: Rats received intraperitoneal injection of the mK_ATP_ inhibitor 5-Hydroxydecanoic (10 mg/kg) 15 min before SPC. 5-HD = 5-Hydroxydecanoic; CHE = Chelerythrine; IPC = ischemic preconditioning; IRI = ischemia-reperfusion injury; SPC = sevoflurane preconditioning; SMA = superior mesenteric artery. * = SMA occlusion for 5 min; # = reperfusion for 5 min.

Two experiments were performed. Experiment one was designed to compare the protection of IPC and SPC in rats with intestinal IR. Rats randomly received one of the following treatments (Fig 1, n = 9 per group): (1) Sham: Rats were subject to laparotomy and isolation of SMA without occlusion; (2) IRI: Rats underwent SMA occlusion for 60 min and reperfusion for 120 min; (3) SPC-sham: Rats were pretreated with 0.5 MAC sevoflurane for 30 min before the sham operation; (4) IPC-sham: Rats underwent intestinal IPC without ischemia; (5) SPC: Rats were pretreated with 0.5 MAC sevoflurane for 30 min before ischemia; (6) IPC: Rats underwent intestinal IPC prior to ischemia. At the end of reperfusion, three milliliters of venous blood samples from the infrahepatic vena cava of each rat was collected to explore the changes in malondialdehyde (MDA), superoxide dismutase (SOD) and tumor necrosis factor-a (TNF-α), and the intestine tissues were collected 10 cm away from the ileocecal junction to evaluate the histopathological changes after hematoxylin-eosin (HE) staining and to detect apoptosis by Terminal-deoxynucleoitidyl Transferase Mediated Nick End Labeling (TUNEL) staining. All rats were sacrificed by exposure to carbon dioxide at the end of the experiment.

Experiment two was designed to investigate whether SPC protects against intestinal IR by the activation of the PKC or mK_ATP_ pathway. The rats received an intraperitoneal injection of the PKC inhibitor Chelerythrine (CHE, 5 mg/kg in DMSO solution) or the mK_ATP_ inhibitor 5-Hydroxydecanoic (5-HD, 10 mg/kg in DMSO solution) 15 min before SPC (Fig 1, n = 9). At the end of reperfusion, the rats were sacrificed, and the intestine tissues were collected 10 cm away from the ileocecal junction to evaluate the histopathological changes after HE staining, measure caspase-3 activity by immunohistochemistry staining and to determine the protein expression of cleaved caspase-3 and Bcl-2 using western blot analysis. All rats were sacrificed at the end of the experiment.

### Anesthesia exposure

Anesthesia exposure was performed as we described before [9]. Briefly, the rats were placed in a chamber with an inflow hose at the bottom and an outflow hose at the top of the chamber. Sevoflurane in a humidified 30% oxygen carrier gas at 2–3 L/min was delivered to the chamber for 30 min using an agent-specific vaporizer (Datex-Ohmeda, Madison, WI). The concentrations of sevoflurane, oxygen and carbon dioxide in the chamber were measured by a gas analyzer (Datex Cardiocap II, Datex-Ohmeda, Madison, WI) via a sensing device placed in the chamber immediately adjacent to the rats. One minimum alveolar concentration (MAC) of sevoflurane is approximately 2.0% in adult rats according to Orliaguet et al [23]. Therefore, 0.5 MAC of sevoflurane is approximately 1% in adult rats. The fresh gas flow rate was adjusted to keep CO_2_ less than 1%. We demonstrated in a previous study that 0.5 MAC of sevoflurane does not cause a severe disruption of circulation and respiration [9]. The rats were placed on a heating pad and under a warming light to maintain body temperature at approximately 37°C. The rats in the sham, IRI, IPC-sham and IPC groups were exposed only to carrier gas.

### Intestinal histopathology

The samples of the ileum were processed as we previously described [9]. The histopathological changes were scored from 0 to 9 using the criteria of Chiu’s method by two independent pathologists who were blinded to the study groups [24] (grade 0: normal mucosa; grade 1: subepithelial Gruenhagen’s space, capillary congestion; grade 2: moderate intestine grand damage; grade 3: extension of subepithelial space with moderate epithelial lifting; grade 4: massive epithelial lifting down sides of villi, few tips denuded; grade 5: denuded villi; grade 6: loss (destruction) of villi, hemorrhage; grade 7: injured crypt layer, hemorrhage; grade 8: necrosis of the entire mucosa and submucosa, hemorrhage; grade 9: transmural necrosis, hemorrhage. A minimum of six randomly chosen fields from each rat were evaluated and averaged to determine the mucosal damage, and then the results from the two pathologists were averaged.

### Detection of apoptosis after intestinal IRI

Apoptosis was detected in the small intestine (jejunum and ileum) with TUNEL staining as we described previously [9, 25]. A TUNEL fluorescent assay was performed using the Dead End TM fluorometric TUNEL system kit (Promega, Madison, WI, USA) according to the instructions provided by the manufacturer. The slides were protected from direct light during the experiment, and Hoechst was used to stain nuclei. TUNEL positive cells in the crypt epithelium were counted in at least six randomly chosen fields with a magnification of ×400 from each rat by two persons who were blinded to the group assignment and the average number of cells was calculated. The density of TUNEL positive cells was calculated by dividing the average number of TUNEL positive cells by the area of a microscopic field.

### Immunohistochemistry for caspase-3

Caspase-3 positive cells were detected using the immunohistochemical method as we described previously [25, 26]. Briefly, sections were incubated with blocking solution containing 10% normal goat serum in 0.1% phosphate buffered saline with 0.1% Tween 20 (PBST) for 1 h at room temperature. The anti-activated caspase-3 primary antibody (1/200, Cell Signaling Technology, Inc Danvers, MA, USA) was then applied in blocking solution and incubated at 4°C overnight. Tissue sections were biotinylated with goat anti-rabbit antibody (1/200, Santa Cruz Biotechnology, Inc., Santa Cruz, CA, USA) in 0.1% PBST for 40 min, followed by incubation with the avidinebiotinylated peroxidase complex (Vectostain ABC-Kit, Vector Lab, Burlingame, CA, USA) for 40 min, colorized with DAB and counterstained with modified hematoxylin. Negative control sections were incubated in blocking solution that did not contain primary antibody. Images were acquired and assessed at 400X using IP lab 7.0 software linked to an Olympus IX70 microscope (Olympus Corporation, Japan) equipped with a Cooke SensiCam camera (Cooke Corporation, Romulus, MI, USA).

### Immunoblotting

Western blotting was performed as we have described previously [25, 27]. Briefly, the protein concentrations of samples were determined using the bicinchoninic acid assay reagent (Pierce Chemical Company, Rockford, IL). Thirty micrograms of each protein sample were subjected to western blot analysis using the following primary antibodies: anti-cleaved caspase-3 at 1:2000 dilution, anti-Bcl-2 at 1:1000 dilution, and anti-β-actin at 1:2000 dilution. All antibodies were purchased from Cell Signaling Technology, Beverly, MA, USA. Images were scanned by an Image Master II scanner (GE Healthcare, Milwaukee, WI, USA) and were analyzed using ImageQuant™ TL software v2003.03 (GE Healthcare, Milwaukee, WI, USA). The band signals of other interesting proteins were normalized to those of the corresponding β-actin and then expressed as fractions of the control sample from the same gels.

### Plasma MDA and SOD activity assay

Venous blood samples were taken from the infrahepatic vena cava after 2 h of reperfusion. The blood samples were centrifuged at 3,500 g for 10 min at 4°C to sediment the erythrocytes. The plasma was removed. MDA activity were measured using the chemical assay kits (Nanjing Jiancheng Bioengineering Institute, Nanjing, China) according to the manufacturer's instructions, and their absorbance was measured at 532 nm by a microplate reader (BioTek, SynergyHT, USA). The results were expressed as micromol per milligram. SOD activity was measured using the chemical assay kits (Nanjing Jiancheng Bioengineering Institute, Nanjing, China) according to the manufacturer's instructions and their absorbance was measured at 450 nm by a spectrophotometer (Ruili, Model VIS-723G, Beijing, China). The results are expressed as unit per milliliter.

### Enzyme linked immunosorbent assays (ELISAs) for TNF-α

The plasma concentrations of TNF-α were determined using commercially available enzyme-linked immunosorbent assay (ELISA) kits (Pierce Biotechnology, Rockford, USA) according to the manufacturer’s procedure. The results are expressed as pictograms per milliliter plasma.

### Statistical analysis

The data of Chiu scores, TUNEL assay, western blots and plasma concentrations of MDA, SOD and TNF-α were normally distributed and had equal variances. They are expressed as the mean ± SD and were analyzed using one-way ANOVA, followed by Bonferroni’s posttest. All possible comparisons between groups were made using Bonferroni’s posttest. The GraphPad Prism 6.0 software was used to conduct the statistical analyses. Statistical significance was accepted at *P* < 0.05.

## Results

### Both SPC and IPC Ameliorated Intestinal IRI

**Fig 2:**
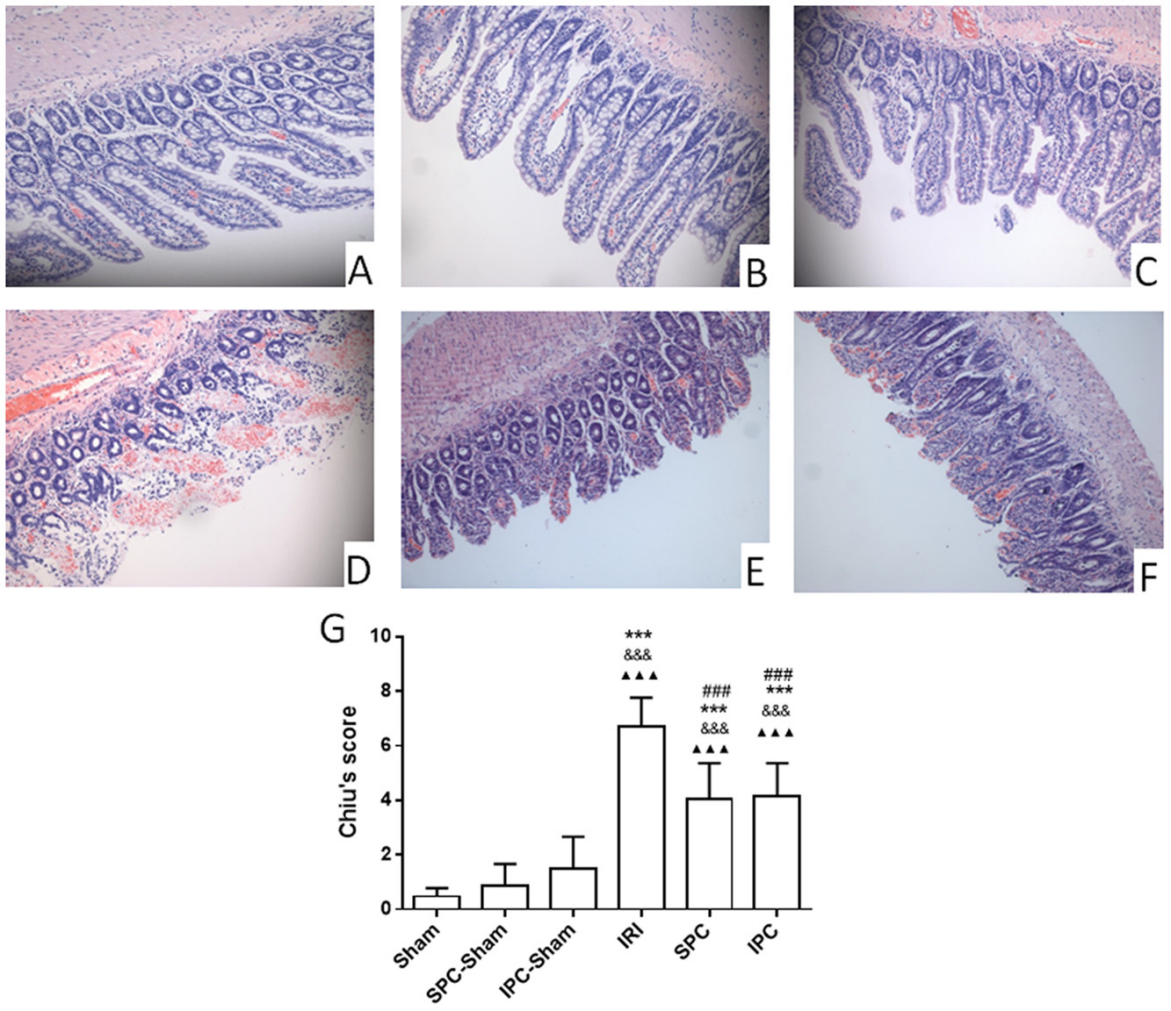
Both SPC and IPC inhibited intestinal IRI. (A-F) Histopathological changes of intestinal mucosa under light microscopy (×200); (G) The evaluation of intestinal injury with Chiu’s scores. In the sham (A) and SPC- sham (B) groups, there were no injuries to the villi and glands, whereas mildly injured villi and glands were observed in the IPC-sham group (C). However, severe intestinal glands injury, mucosa villi disintegration or edema, increased gap of epithelial cells and severe hemorrhage were observed in the IRI group (D). In the SPC (E) and IPC (F) groups, the damageto intestinal villi and glands was much slighter than that in the IRI group. The Chiu’s score data are expressed as the mean ± SD, n = 9. Results were compared using one-way ANOVA with Bonferroni’s posttest. *** *P < 0*.*001* vs. the Sham group, * *P < 0*.*05* vs. the Sham group, ^&&&^ *P* < 0.001 vs. SPC-Sham. ^▲▲▲^ *P* < 0.001 vs. IPC-Sham, ^###^ *P* < 0.001 vs. the IRI group. IPC = ischemic preconditioning; IRI = ischemia-reperfusion injury; SPC = sevoflurane preconditioning.

The morphology in the intestinal mucosa was normal in rats of the sham and SPC-sham groups (Fig 2A and 2B), whereas mildly injured villi and glands were observed in the IPC-sham group (Fig 2C). The rats subjected to ischemia-reperfusion (IR) suffered severe injuries as shown by sloughing of villous tips, lifting of the epithelium from the lamina propria, apparently injured glands and telangiectasis (Fig 2D). However, both SPC and IPC profoundly reduced mucosal injury caused by intestinal IR, as we found less sloughing of villi tips, mildly injured glands and ameliorated telangiectasis in the tissue samples (Fig 2E and 2F). The injury severity was evaluated by Chiu’s scoring (Fig 2G). Compared with the sham rats, intestinal IR remarkably increased Chiu’s score (*P < 0*.*001*, vs. Sham, SPC-Sham and IPC-Sham). However, in the SPC and IPC groups, Chiu’s scores were dramatically attenuated by 39.74% and 38.08% respectively, compared with those in the IRI group (*P < 0*.*001; P < 0*.*001*; vs. IRI, respectively). The Chiu’s scores of SPC were similar to those of IPC, and there was no significant difference in Chiu’s scores between the SPC and IPC groups (*P > 0*.*999*). Compared with the sham group, the Chiu’s scores in the SPC-Sham and IPC-Sham groups showed no significant difference (*P* > 0.999, *P* = 0.7090).

### Both SPC and IPC Reduced Small Intestinal Apoptosis after IRI

**Fig 3:**
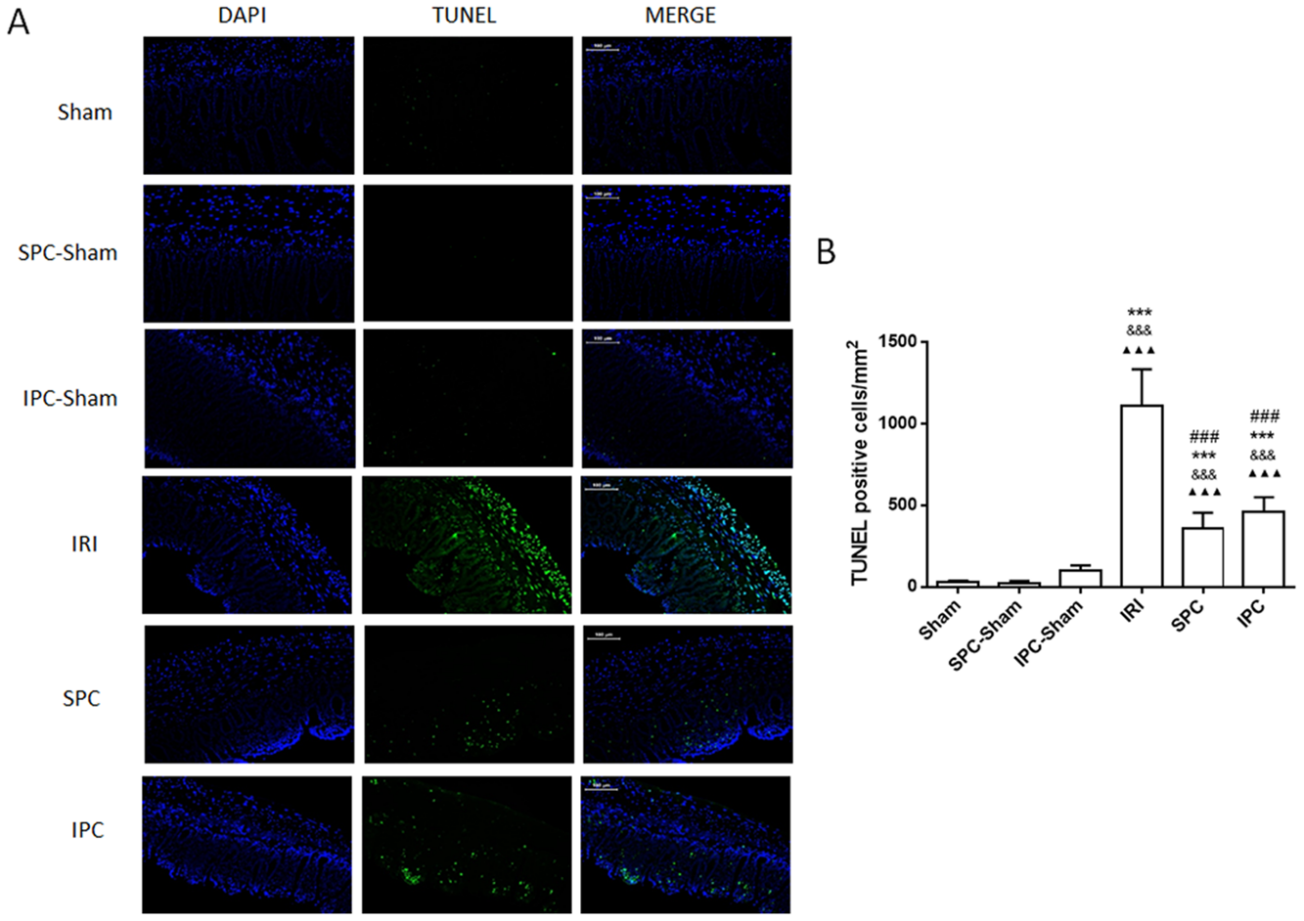
Both SPC and IPC inhibited the increase in IRI-induced TUNEL positive cells in the intestine of rats. (A) Representative images of TUNEL fluorescent staining in the intestinal mucosa (×200) (scan bar = 100 μm). Green staining indicates TUNEL-positive cells, blue staining indicates nuclear. (B) Quantification of TUNEL positive cells in the intestinal mucosal. Data are expressed as the mean ± SD, n = 9. Results were compared using one-way ANOVA with Bonferroni’s posttest. *** *P < 0*.*001* vs. Sham, ^&&&^ *P* < 0.001 vs. SPC-Sham. ^▲▲▲^ *P* < 0.001 vs. IPC-Sham, ^###^ *P < 0*.*001* vs. IRI. IPC = ischemic preconditioning; IRI = ischemia-reperfusion injury; SPC = sevoflurane preconditioning.

We evaluated intestinal apoptosis by TUNEL staining (Fig 3A). Compared with the sham rats, the rats after intestinal IR showed an increase in TUNEL-positive cells per mm^2^ by approximately 43-fold in the small intestine (*P < 0*.*001*, vs. Sham, Fig 3B). In contrast, both SPC and IPC treatment significantly reduced the number of apoptotic cells by 67.63% and 58.77%, respectively, compared with the rats after intestinal IR (*P < 0*.*001; P < 0*.*001*, vs. IRI, respectively, Fig 3B). There was no significant difference between the SPC and IPC groups (*P = 0*.*9144*). The apoptotic cells in the SPC-sham and IPC-sham groups also showed no significant difference compared with the sham group (*P > 0*.*999; P > 0*.*999*, respectively).

### Both SPC and IPC Reduced Plasma MDA Activity

**Fig 4:**
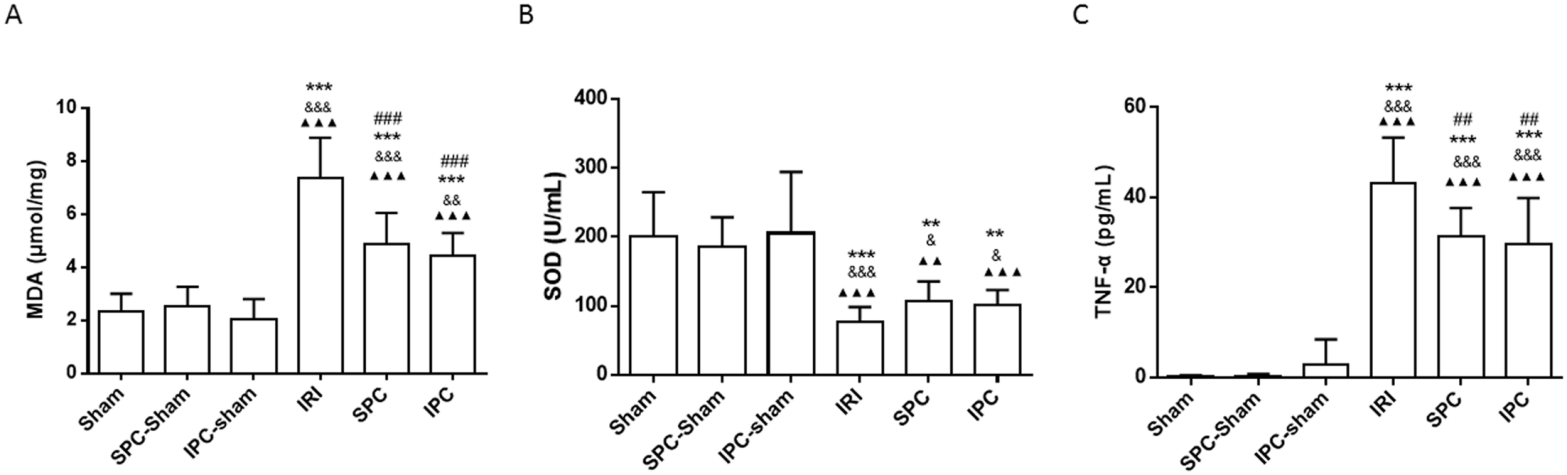
Both SPC and IPC attenuated the increased plasma MDA and TNF-α induced by IRI, but did not significantly change the level of SOD. (A) Quantification of the plasma MDA level; (B) Quantification of the plasma SOD level; (C) Quantification of the plasma TNF-α level. Data are expressed as the mean ± SD, n = 9. Results were compared using one-way ANOVA with Bonferroni’s posttest. ** *P < 0*.*01*, *** *P < 0*.*001* vs. Sham; ^&^ *P* < *0*.*05*, ^&&^ *P* < *0*.*01*, ^&&&^ *P* < 0.001 vs. SPC-Sham; ^▲▲^ *P* < *0*.*01*, ^▲▲▲^ *P* < 0.001, vs. IPC-Sham; ^##^ *P < 0*.*01*, ^###^ *P < 0*.*001* vs. IRI. IPC = ischemic preconditioning; IRI = ischemia-reperfusion injury; SPC = sevoflurane preconditioning.

We evaluated oxidative stress by plasma MDA and SOD activity shown in Fig 4A and 4B. Intestinal IR significantly increased the MDA level compared with the sham control (*P < 0*.*001*, vs. Sham). Both SPC and IPC treatments partly inhibited the intestinal IR—induced increase of MDA level (*P < 0*.*001; P < 0*.*001*, vs. IRI, respectively). The MDA level in the SPC group was similar to that of the IPC group (*P > 0*.*999*). There was no difference among the three sham groups (all *P > 0*.*999*).

The rats in the IRI group had a lower SOD level than the sham, SPC-sham and IPC-sham rats (all *P < 0*.*001*). Both SPC and IPC treatments slightly increased the SOD level, but there was no significant difference compared with rats in the IRI group (*P > 0*.*999; P > 0*.*999* vs. IRI, respectively).

### Both SPC and IPC Reduced Plasma TNF-α concentration

The concentrations of TNF-α were evaluated using specific ELISAs and are displayed in Fig 4C. Intestinal IR resulted in a significant increase in plasma TNF-α compared with that in the sham control group (IRI: 43.24±10.05 pg/ml vs. Sham: 0.13±0.33 pg/ml, *P < 0*.*001*), whereas SPC and IPC significantly reduced TNF-α levels by 27.66% and 31.57%, respectively (SPC: 31.28±6.36 pg/ml; IPC: 29.59±10.28 pg/ml; vs. IRI: 43.24±10.05 pg/ml, *P = 0*.*0079; P = 0*.*0015*, respectively). There was no significant difference between the SPC and IPC groups (*P > 0*.*999*) and among the three sham groups (*all P > 0*.*999*).

### PKC and mK_ATP_ Inhibitors Reversed the Protection Induced by SPC

**Fig 5:**
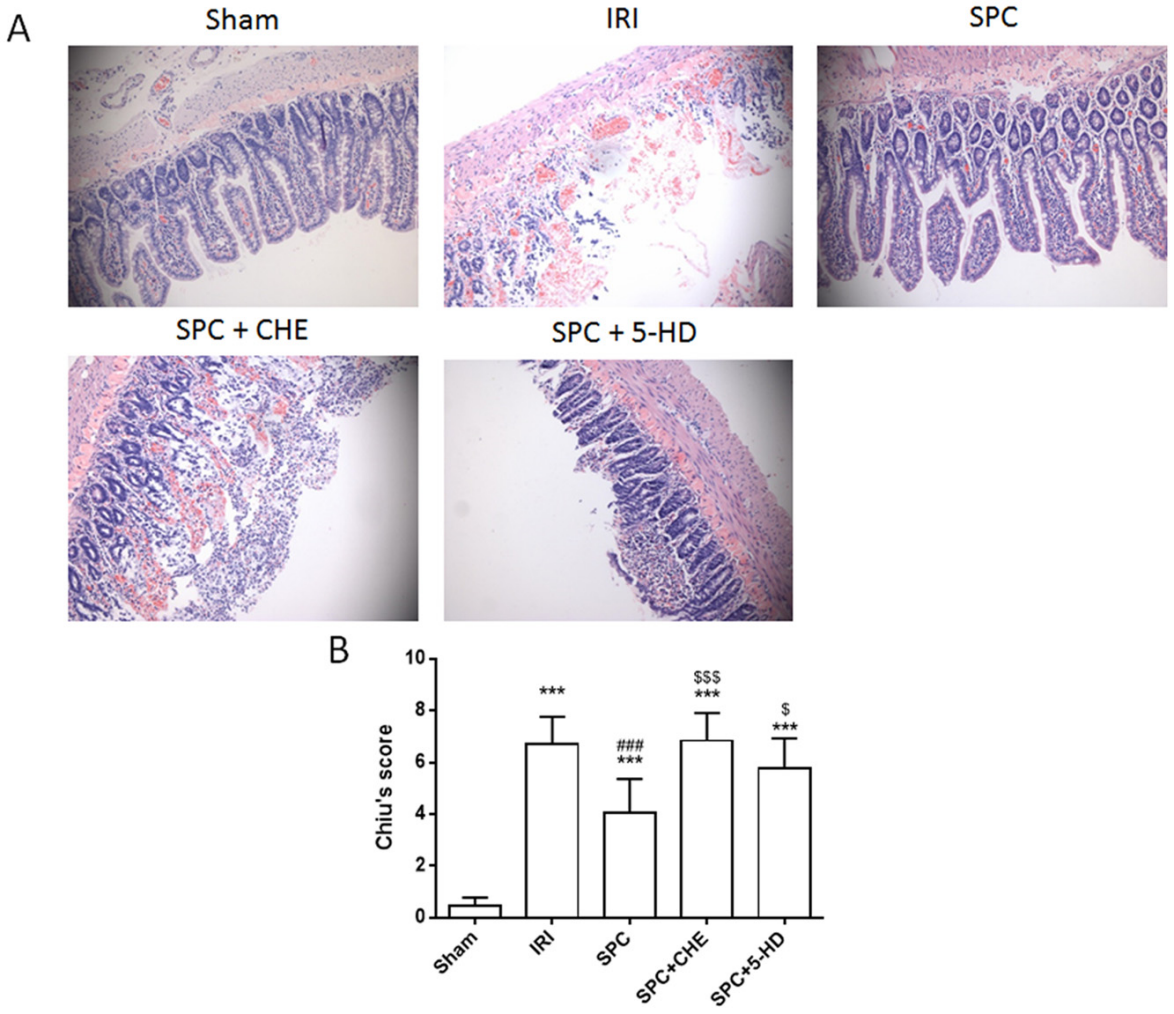
CHE and 5-HD inhibited the protective effect of SPC against intestinal IRI. (A) Morphologic changes of intestinal mucosa under light microscopy (×200); (B) The evaluation of intestinal injury with Chiu’s scores. The Chiu’s score data are expressed as the mean ± SD, n = 9. Results were compared using one-way ANOVA with Bonferroni’s posttest. *** *P < 0*.*001* vs. Sham, ^###^ *P < 0*.*001* vs. IRI, ^$^ *P < 0*.*05*, ^$$$^ *P < 0*.*001* vs. SPC. 5-HD = 5-Hydroxydecanoic; CHE = Chelerythrine; IRI = ischemia-reperfusion injury; SPC = sevoflurane preconditioning.

Consistent with the previous data, intestinal IR led to severe damage to the intestinal mucosa (Fig 5, *P < 0*.*001*, vs. Sham). SPC alleviated small intestinal mucosal injury, as the Chiu’s scores were significantly decreased compared to those of the rats with intestinal IR (Fig 5, *P < 0*.*001*). However, both the PKC inhibitor CHE and mK_ATP_ channel inhibitor 5-HD reversed the protection induced by SPC and increased Chiu’s scores by 69.31% and 42.33%, respectively, compared with the SPC rats (Fig 5; *P < 0*.*001; P = 0*.*0108*, respectively).

### Both PKC and mK_ATP_ Pathways Were Involved in the Protection of SPC by Inhibiting Apoptosis

**Fig 6:**
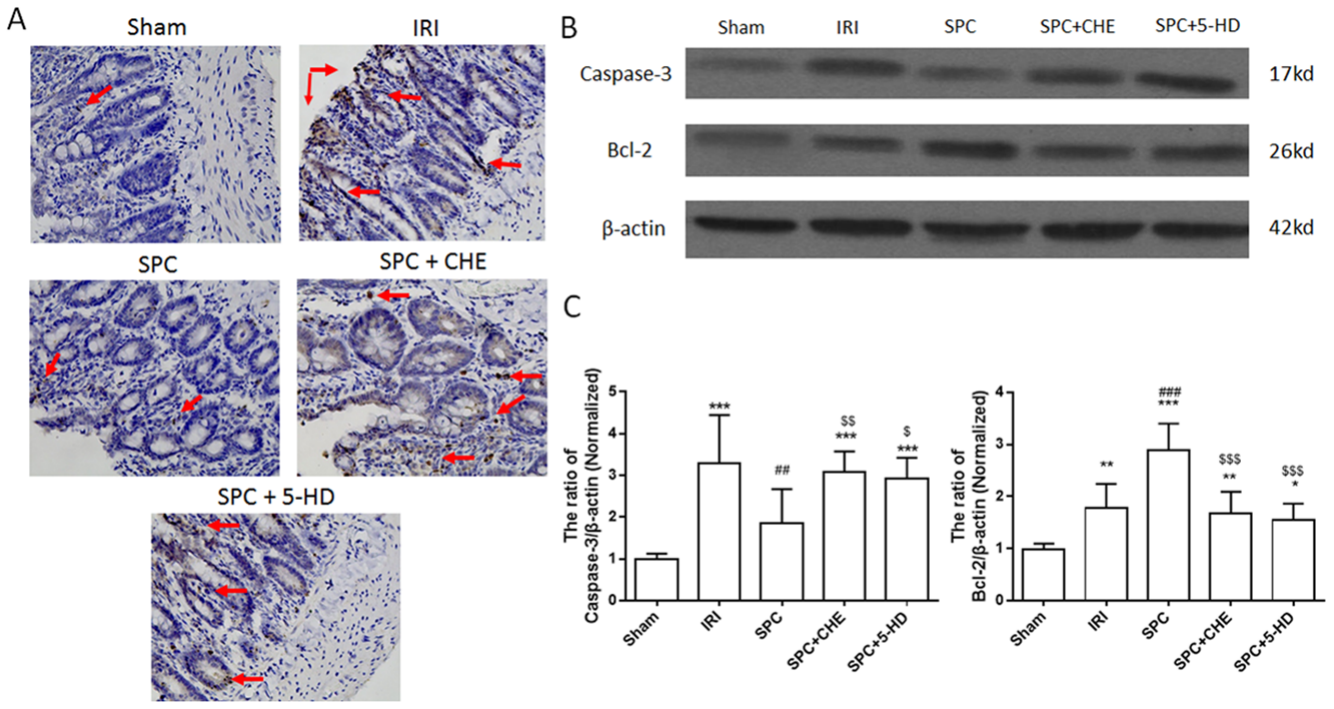
CHE and 5-HD reversed the inhibition of SPC on intestinal apoptosis induced by intestinal IRI in rats. (A) Representative immunohistochemical images of cleaved caspase-3 in the intestinal mucosa (×400), caspase-3 positive cells are stained dark brown under light microscopy shown by the arrows; (B) Representative western blots of cleaved caspase-3 and Bcl-2; (C) The quantitative analysis of Bcl-2 and caspase-3. Data are expressed as the mean ± SD, n = 9. Results were compared using one-way ANOVA with Bonferroni’s posttest. * *P < 0*.*05*, ** *P < 0*.*01*, *** *P < 0*.*001* vs. the Sham; ^###^ *P < 0*.*001* vs. IRI; ^$^ *P < 0*.*05*, ^$$^ *P < 0*.*01*, ^$$$^ *P < 0*.*001* vs. SPC. 5-HD = 5-Hydroxydecanoic; CHE = Chelerythrine; IRI = ischemia-reperfusion injury; SPC = sevoflurane preconditioning.

Immunohistochemistry of active caspase-3 was conducted to provide an overview of cell apoptosis in intestinal mucosa. As shown in Fig 6A, caspase-3 positive cells are stained dark brown under light microscopy. Compared with the sham control, the rats subjected to intestinal IR demonstrated a large quantity of caspase-3 positive cells distributed throughout the mucosa, including the villi, glands and stratum basal. SPC decreased the number of caspase-3 positive cells, whereas CHE or 5-HD reversed the protection of SPC and increased caspase-3 positive cells compared with the SPC rats. The expression of cleaved caspase-3 protein in intestinal tissues was also detected by western blot in a quantification analysis (Fig 6B and 6C). Intestinal IR significantly increased caspase-3 activation by 2.2-fold compared with the sham group (*P < 0*.*001*, vs. Sham). SPC significantly reduced intestinal IR-induced caspase-3 expression by 40.63% (*P 0*.*0012*, vs IRI), whereas CHE or 5-HD recovered caspase-3 expression by 57.89% and 47.37%, respectively (*P = 0*.*0068*, *P = 0*.*0274*, vs. SPC, respectively).

Furthermore, the expression of Bcl-2 protein was also used to evaluate apoptosis in the intestinal mucosa (Fig 6B and 6C). Intestinal IR slightly up-regulated the expression of Bcl-2 protein (*P = 0*.*0013*, vs. sham), which was significantly increased by 2-fold in the SPC group, compared with the sham control (*P < 0*.*001*, vs. sham). Both CHE and 5-HD reduced the expression of Bcl-2 compared with the SPC rats (*P < 0*.*001; P < 0*.*001*, vs. SPC, respectively). There was no significant difference between pretreatment with CHE and 5-HD inhibitor (*P > 0*.*999*).

## Discussion

The present study demonstrated that 0.5 MAC SPC provides similar protective effects as IPC by preventing intestinal IR-induced histopathological changes, intestinal mucosa apoptosis, MDA activation and expression of TNF-α. Additionally, the protection of SPC is abolished by the PKC inhibitor CHE and the mK_ATP_ channel blocker 5-HD. These observations indicate that the activation of the PKC and mK_ATP_ signaling pathways may be involved in the mechanisms by which SPC reduces intestinal IRI.

Intestinal ischemia is a major cause of multiple organ failure and has been described as a consequence of low splanchnic blood flow after liver transplantation, cardiac arrest, shock or cardiac surgery [1, 2]. IPC refers to a phenomenon in which exposure of a tissue to brief periods of ischemia protects them from the deleterious effects of prolonged IRI. IPC of the intestine was first described by Hotter et al in 1996 [4], and subsequent studies have confirmed that it is a potential strategy among various therapeutic modalities [3, 5–8]. However, IPC has limited application in the clinic due to its invasive nature and the unpredictability of intestinal ischemia. Increasing evidence has elucidated that sevoflurane prevents ischemia-reperfusion injury of multiple organs, including the brain, heart and lung [10, 12, 20, 27, 28, 29]. Our recent study also proved that 0.5 MAC SPC provides the best protective effects against intestinal IRI among all exposure strategies [9]. In the current study, we compared the protective potency of SPC with IPC. Our pathology results showed that SPC and IPC equally reduced the Chiu’s score in rats with intestinal IR, suggesting that SPC and IPC ameliorate intestinal pathological injury to the same degree.

The mechanisms by which IPC confers protection in the intestine includes stimulation of adenosine A1 receptors [5], activation of NO synthesis [4, 6], inhibition of oxidative stress [3], abolishment of cytokines expression [7] and reduction of apoptosis [8]. Our present results showed that IPC reduced apoptosis in the intestinal mucosa, inhibited oxidative stress by decreasing the plasma level of MDA and diminished the inflammatory response by decreasing the concentration of TNF-α, which were consistent with previous studies [7, 8]. Moreover, SPC also ameliorated the above measurement indicators in the same way that IPC did, indicating that some mechanisms of SPC are the same as IPC. Oxidative stress reflects an intracellular imbalance of over-production of reactive oxygen species (ROS) such as free radicals and low generation of antioxidants such as SOD. During reperfusion, the intestine is the richest initial source of free radicals—superoxide anion (O^-2^) and hydrogen peroxide (H_2_O_2_). Under physiological conditions, the damaging effects of O^-2^ are prevented by SOD, which converts O^-2^ to H_2_O_2_. However, during reperfusion of ischemic tissues, these natural defenses may be overcome. ROS causes biological damage by stimulating the free chain reaction known as lipid peroxidation with end products including MDA and 4-hydroxynonenal (HNE) and finally causes organelle and cell disruption [30–32]. In the current study, we demonstrated that SPC attenuated the production of MDA and TNF-α identically to IPC, indicating that SPC decreases intestinal IRI by inhibiting oxidative stress and inflammatory response. Gan et al recently reported that SPC significantly down-regulates myeloperoxidase (MPO) activities, intercellular cell adhesion molecule-1 (ICAM-1) expression, and interleukin-6 (IL-6) concentrations, further suggesting that inhibiting neutrophil sequestration and the subsequent systemic inflammation may be potential mechanisms by which SPC attenuates intestinal IRI [33]. Although intestinal IR dramatically suppressed plasma SOD activity, neither IPC nor SPC significantly increased SOD activity in our current study. This finding was not consistent with Ferencz’s study in which IPC significantly recovered SOD activity [3, 34]. The main reasons may be the difference in species, tissue samples and experimental model. They measured the SOD activity of intestinal tissue samples in dogs undergoing small-bowel autotransplantation, whereas in this current study we detected the plasma SOD activity in rats.

Oxidative stress and inflammatory response are very relevant. Nitric oxide (NO) is considered a key factor in connecting the generation of free radicals and the secretion of inflammatory mediators by endothelial cells. NO has many beneficial effects in the intestine, such as scavenging oxygen free radicals. Whereas NO is absent during the early phase of reperfusion, the O^-2^ produced will undergo spontaneous dismutation to form H_2_O_2_. H_2_O_2_ formed in this way can promote the activation of phospholipase A_2_ (PLA_2_) and lead to the accumulation of proinflammatory mediators such as platelet activating factor (PAF) and leukotrienes (LTB4). These changes can lead to the activation of transcription nuclear factor kappa B (NF–κB), which induces the expression of cytokine (TNF-α and β, interleukins) and adhesion molecule (ICAM-1, VCAM-1, P-selectin, E-selectin) to result in systemic inflammation [35]. The activation of endothelial NO synthase (eNOS), attenuation of NF–κB activation and down-regulation of NF–κB dependent inflammatory gene expression play important roles in the protective mechanism of SPC against acute myocardial IRI [36, 37]. It is possible that SPC also inhibits the intestinal inflammatory response by activating eNOS and suppressing the NF–κB pathway; these theories need further investigation.

A previous study demonstrated that the K_ATP_ channel blocker glibenclamide protects intestinal IRI by inhibiting the intestinal IR-associated neutrophil accumulation and the increase in TNF-α and IL-6 levels, suggesting the role of the K_ATP_ channel in mediating the neutrophil flux and inflammatory response during intestinal IRI [38]. In contrast to glibenclamide, which is a popular K_ATP_ inhibitor that acts on both mitochondrial and plasma membrane channels, 5-HD is considered selective for mitochondrial K_ATP_ channels [39]. It has been reported that opening of the mKATP channels mediated neuroprotection as well as cardioprotection of sevoflurane preconditioning or postconditioning by applying 5-HD [40, 41]. Studies on the expression of the mKATP channel subunit Kir6.2 have also confirmed the mKATP channel contributes to neuroprotection of sevoflurane postconditioning after focal cerebral ischemia in rats [42]. Opening of the mKATP channels is associated with an uptake of potassium in the mitochondrial matrix, which can maintain the mitochondrial matrix volume by limiting Ca^2+^ accumulation [43]. This reduction in mitochondrial Ca^2+^ accumulation would prevent, during reperfusion, the opening of the mitochondrial permeability transition pore (MPTP) known to inhibit oxidative phosphorylation and facilitate the release of proapoptotic proteins [43, 44]. It has been proven that the mKATP channel blocks the calcium-induced MPTP opening in ischemia and anesthetic-induced myocardial preconditioning [45]. Our present results showed that the inhibition of mKATP channels with 5-HD abolished the protection conferred by sevoflurane preconditioning by increasing intestinal histopathological injury and apoptosis, suggesting that opening of mKATP channels may be critical for sevoflurane preconditioning. Hanley et al suggests another mechanism of 5-HD with K(ATP) channel-independent targets using submitochondrial particles isolated from pig heart, showing that 5-HD can be metabolized to its CoA derivative (5-HD-CoA) and then enters the β-oxidation pathway [46]. Therefore, it is also likely that the inhibitory effect of 5-HD on preconditioning might result from both metabolic effects and pharmacological modulation of channel activity. In our study, the application of 5-HD inhibited the SPC induced increase of BCL-2 and decrease of caspase-3, which indicated 5-HD may active the mitochondrial apoptosis pathway, and we did not measure mitochondrial membrane potential or the expression of the mK_ATP_ channel subunit Kir6.2 or assess any of its off-target effects. Therefore, we could not confirm that the inhibitory effect of 5-HD on SPC is direct by inhibiting mK_ATP_ channels or metabolic effects.

IR-induced leukocyte rolling and adhesion is critically dependent on the expression of P-selectin in endothelial cells. PKC antagonist prevents the protective effect of IPC in intestinal mucosa and also reverses the limit of IPC on IR-induced P-selectin expression [21, 47]. Our present results showed that the intestinal protection of SPC was also abolished by pretreatment with non-selective PKC antagonists CHE, demonstrating that the protection of SPC against intestinal IRI is also dependent on the activation of the PKC pathway. Except for the effect of anti-inflammation, PKC participates in the precise regulation of cellular metabolism and apoptosis through changing the status of mitochondria. After PKC is activated and translocated to the cytomembrane, it triggers the phosphorylation of multiple intracellular factors, and leads to the activation of the mK_ATP_ channel [48, 49]. Opening of the activated mK_ATP_ channel induces blockage of the MPTP and then inhibition of apoptosis [43–45]. Some studies have shown that delayed MPTP opening is mediated by the PKC-dependent pathway in rats pretreated with anesthetics [50]. Our present results showed that intestinal IR significantly activated the expression of caspase-3 and slightly increased the expression of anti-apoptosis protein Bcl-2, which suggests that some anti-apoptotic mechanisms are activated during intestinal IR. These findings were consistent with our previous study which showed that intestinal IR slightly increased the activity of anti-apoptosis protein Akt [9]. However, SPC significantly increased the expression of Bcl-2 and inhibited activation of casase-3. PKC antagonist CHE abolished the protection of SPC by increasing intestinal histopathological injury and apoptosis and reversing Bcl-2 and casase-3 protein changes, suggesting that the PKC pathway may be involved in the protection of SPC against intestinal IRI.

It has been proven that the opening of the mK_ATP_ channel occurs downstream of PKC-epsilon activation in the mechanism of preconditioning [51, 52], whereas recent studies suggest that PKC epsilon translocation may be a downstream event of mK_ATP_ [20, 53]. It is possible that there is feedback regulation between PKC and mK_ATP_. Studies on cardioprotection induced by IPC showed that the activation of PKC leads to the opening of the mK_ATP_ channel to induce potassium influx, which triggers modest ROS formation and then activates PKC in turn [48, 54]. Whether there is a feedback regulation between PKC and mK_ATP_ in SPC against intestinal IRI needs further investigation. In the present study, CHE and 5-HD abolished SPC—induced protection to different degrees. CHE completely abolished the improvement in histology and apoptosis induced by SPC in intestinal mucosa, whereas 5-HD only partially reversed them. Because CHE is a nonselective PKC inhibitor, it may block more PKC isoforms than selective PKC inhibitors. In addition to the anti-inflammation and anti-apoptosis effects, PKC has more biological functions. Liu ZG et al reported that after isoflurane inhalation, the activated PKC increased the expression of vascular endothelial growth factor (VEGF), which may provide protection by promoting cell proliferation and immigration [55]. These findings may partly explain why CHE completely abolished SPC—induced protection.

There are several limitations in our study. First, we did not observe the effects of SPC on the expression of PKC isoforms, and we did not directly assess the changes of the mKATP channel to confirm the target effect of 5-HD. Additional experiments are needed to clarify which PKC isoforms play an important role and whether the inhibitory effect of 5-HD on SPC directly inhibits mKATP channels in the protection of SPC against intestinal IRI. Additionally, we only measured TNF-α to evaluate the inflammatory response, and we did not observe the effects of PKC and mK_ATP_ inhibitor on oxidative stress and the inflammatory response after SPC. Whether activation of PKC and mK_ATP_ is associated with SPC-induced inhibition of oxidative stress and the inflammation response needs further investigation.

In conclusion, SPC in clinically relevant concentrations provided similar protective effects as IPC by preventing intestinal IR-induced apoptosis, oxidative stress and the inflammatory response. Activation of PKC and mK_ATP_ pathways may partly participate in the protective effect of SPC. Because sevoflurane preconditioning is non-traumatic and easy to control, it will be an effective alternative method in addition to IPC in facing the challenge of intestinal IR.

## Supporting Information

S1 File The ARRIVE Guidelines Checklist. (PDF)
